# Acupuncture Effect and Mechanism for Treating Pain in Patients With Parkinson's Disease

**DOI:** 10.3389/fneur.2019.01114

**Published:** 2019-10-22

**Authors:** Shao-Wen Yu, Sung-Han Lin, Chih-Chien Tsai, Kallol Ray Chaudhuri, Yu-Chieh Huang, Yu-Sheng Chen, Bo-Yan Yeh, Yih-Ru Wu, Jiun-Jie Wang

**Affiliations:** ^1^Division of Acupuncture and Chinese Traumatology, Department of Traditional Chinese Medicine, Chang Gung Memorial Hospital, Linkou, Taiwan; ^2^Medical Imaging and Radiological Sciences, Chang Gung University, Taoyuan, Taiwan; ^3^Parkinson Foundation International Centre of Excellence, King's College Hospital and Kings College, London, United Kingdom; ^4^Department of Medical Imaging and Intervention, Chang Gung Memorial Hospital, Taoyuan, Taiwan; ^5^Graduate Institute of Clinical Medical Science, College of Medicine, Chang Gung University, Taoyuan, Taiwan; ^6^Department of Neurology, Chang Gung Memorial Hospital Linkou Medical Center and College of Medicine, Chang Gung University, Taoyuan, Taiwan; ^7^Department of Medical Imaging and Intervention, Chang Gung Memorial Hospital, Keelung, Taiwan; ^8^Healthy Aging Research Center, Chang Gung University, Taoyuan, Taiwan

**Keywords:** pain, Parkinson's disease, acupuncture, rs-fMRI, functional connectivity

## Abstract

Non-motor symptoms of Parkinson's disease (PD) have been receiving increasing attention. Approximately half of patients with PD have experience PD-related pain. We investigated the effect and mechanism of acupuncture in patients with PD who have pain. PD patients with pain were divided into acupuncture group and control group. Nine patients completed acupuncture treatment; seven patients who received only an analgesic agent underwent resting-state functional magnetic resonance imaging (rs-fMRI) twice. fMRI was performed to evaluate the functional connectivity of the brain regions. After treatment, a decrease in total scores on the King's Parkinson's Disease Pain Scale (KPPS) and Unified Parkinson's Disease Rating Scale was observed in the acupuncture group (−46.2 and −21.6%, respectively). In the acupuncture group, increased connectivity was observed in four connections, one in the left hemisphere between the middle temporal gyrus (MTG) and precentral gyrus, and three in the right hemisphere between the postcentral gyrus and precentral gyrus, supramarginal gyrus and precentral gyrus, and MTG and insular cortex. A significant correlation was noted between the changes in functional connectivity and KPPS. The involved connection was between the left middle frontal gyrus and the right precentral gyrus (*R* = −0.698, *P* = 0.037). Acupuncture could relieve pain in PD patients by modulating brain regions related to both sensory-discriminative and emotional aspects. The present study might increase the confidence of users that acupuncture is an effective and safe analgesic tool that can relieve PD-related pain.

## Introduction

The non-motor symptoms (NMS) (1) of Parkinson's disease (PD) have gained increasing attention because of their heavy burden on patients and patient caregivers (2) and are now known to be integral to the concept of PD from prodromal to the palliative stage. The prevalence of NMS in PD is up to 98.6%, of which 40–90% of patients have pain. Patients might not report symptoms of pain to their physicians because most patients are unaware that such symptoms can be linked to PD (3). Insufficient treatment for these symptoms may affect patients' quality of life or even lead to hospitalization (3).

Pain in PD might be a consequence of motor fluctuations, dystonic muscle contraction, deep visceral pain, and musculoskeletal pain (1–4). It can be influenced by factors such as age, sex, depression, and disease severity or duration (2, 3, 5). As the disease progresses, patients might have a reduced pain threshold compared with healthy individuals (4) and thus might experience pain more easily, especially musculoskeletal pain. The general management of pain in patients with PD includes dopaminergic therapy, use of anti-inflammatory agents, physical therapy, and surgery (6). The pain may be treated with levodopa, but unfortunately this treatment method does not always lead to improvement (7). As a result, these treatments lead to additional financial burden in medical treatment.

Acupuncture has long been used clinically for pain relief (8), such as migraine pain, lower back pain, chronic pain, and cancer pain. It has been practiced worldwide as an integrative medical therapy that stimulates the meridian or energy-carrying channel through an acupuncture point (an “acupoint”) to correct imbalances in the human body. However, reports on PD-related pain relief are still lacking. The mechanism of the analgesic effect of acupuncture is still unclear.

Pain is a complex experience including not only sensory but also emotional dimensions. Resting-state functional magnetic resonance imaging (rs-fMRI) (9) is an effective and non-invasive method for recording brain functional activity and exploring the underlying neural mechanisms of pain through blood-oxygen-level–dependent effects (10). This brain imaging technique has revealed that the pain matrix consists mainly of the thalamus, amygdala, insular cortex, supplementary motor area, prefrontal cortex, anterior cingulate cortex, and periaqueductal gray (11). The hypothesis of our study is that the effect of acupuncture might be achieved through modulation of the activated cortical and subcortical networks (e.g., limbic, cerebellar, and brainstem). The aim of the present study was to investigate the mechanism of acupuncture in treating patients with PD who have pain by measuring the changes in brain functional connectivity with rs-fMRI.

## Materials and Methods

### Participants

The present study was approved by the ethics committee of the Chang Gung Medical Foundation Institutional Review Board (approval number: 201600710A3C101). Written informed consent was obtained from each participant after full explanation of the details of the study procedures. The study complied with the principles of the Declaration of Helsinki.

Patients with PD who had pain were consecutively enrolled at the outpatient clinic of the Neurology Department of Chang Gung Memorial Hospital in Taiwan. The diagnosis of idiopathic PD was made according to the UK Brain Banks Network criteria (12). The inclusion criteria were (1) diagnosis of idiopathic PD and (2) a total score of >0 on the King's Parkinson's Disease Pain Scale (KPPS) (13). Patients were divided into two groups according to whether they received acupuncture therapy (acupuncture group) or did not (control group). The exclusion criteria were as follows: (1) Mini Mental State Examination (MMSE) score of <24, in order to ensure that the participants could express their feelings accurately; (2) previous acupuncture treatment within 3 months; (3) diagnosis of disorders causing pain unrelated to PD (e.g., postoperative pain); (4) presence of conditions incompatible with acupuncture, such as bleeding, coagulation disorders, or skin infections; and (5) general magnetic resonance imaging (MRI) exclusion criteria.

The following assessments were used to evaluate patients' clinical status: (1) KPPS (13), which is the most recently developed scale for evaluating pain in patients with PD; (2) visual analog scale (VAS) (14); (3) Beck Depression Inventory II (BDI-II) (15); (4) Parkinson's Disease Sleep Scale 2 (PDSS-2) (16); (5) 39-item Parkinson's Disease Questionnaire (PDQ-39) (17); and (6) Unified Parkinson's Disease Rating Scale (UPDRS) (18); and (7) MMSE (19). The patients were divided into two groups according to their wish. If they expressed their wish to receive acupuncture therapy, they would be assigned to the acupuncture group. If they preferred not, they would be enrolled as control upon entering the study. The potential pervious acupuncture effect is eliminated by excluding patients who had received previous acupuncture treatment within 3 months. The demographic features are summarized in Table 1.

**Table 1:** Demographic and clinical features in baseline measurement.

	**Acupuncture**	**Control**	**Significance**
Number of patients	9	7	
Age	60.7 ± 6.3	70.4 ± 8.2	*P* = 0.042
Gender (M/F)	4/5	5/2	
Years of disease	9.4 ± 4.1	9.7 ± 5.4	NS
LEDD	708 ± 311.6	732 ± 370.6	NS
**Disease-specific assessment**			
UPDRS Total	35.7 ± 14.2	36.6 ± 11.8	NS
UPDRS I	2.0 ± 1.7	2.9 ± 1.8	NS
UPDRS II	10.8 ± 4.4	11.7 ± 5.4	NS
UPDRS III	18.2 ± 5.5	20 ± 5	NS
UPDRS IV	4.7 ± 5.1	2.0 ± 2.6	NS
**Cognitive and psychological assessment**			
BDI-II	9.2 ± 5.5	11.1 ± 5.0	NS
PDSS-2	15.7 ± 9.3	8.9 ± 4	NS
PDQ-39	30.8 ± 18.4	31.3 ± 14.5	NS
MMSE	28.4 ± 1.3	26.6 ± 2.2	NS
VAS	5.3 ± 2.6	5.1 ± 3.0	NS
KPPS	21.9 ± 19.8	7.1 ± 5.4	NS

*LEDD, Levodopa Equivalent Dose; UPDRS, Unified Parkinson's Disease Rating Scale; BDI-II, Beck Depression Inventory II; PDSS, Parkinson's Disease Sleep Scale; PDQ, Parkinson's Disease Questionnaire; MMSE, Mini-Mental State Examination; VAS, visual analog scale; KPPS, King's Parkinson's Disease Pain Scale; NS, No significance*.

### Study Procedure

All patients underwent evaluations and their first rs-fMRI examination at baseline; patients in the treatment group and control group underwent the second evaluation after acupuncture treatment and 10–14 weeks after the first examination, respectively.

All patients had maintained the same antiparkinsonian medications during the study. Analgesic agents were prescribed according to usual clinical routine. Non-steroidal anti-inflammatory drug (NSAID) is the major analgesic agent in our patients. During this study, they kept their analgesic agent as tolerable, and routine analgesic agent would be added if needed. However, no patient asked for more analgesic medication during the whole study in both groups.

**Figure 1:**
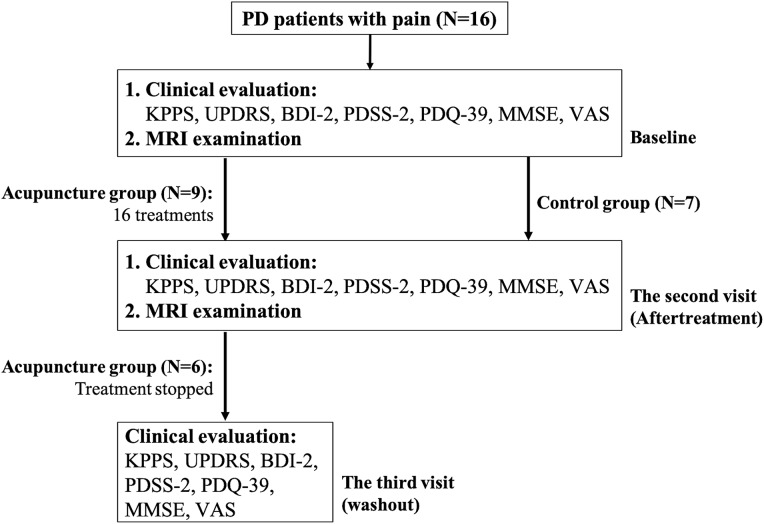
Flow chart of the current study. Patients with PD who had pain were consecutively enrolled and were divided into two groups according to their wish. If they expressed their wish to receive acupuncture therapy, they would be assigned to the acupuncture group. If they preferred not, they would be enrolled as control upon entering the study. All patients underwent clinical evaluations and their first MRI examination at baseline; patients in the treatment group and control group received the second clinical evaluation and MRI examination after 16 acupuncture treatments and 10–14 weeks after the first examination, respectively. The patients in the acupuncture group underwent the third evaluation, three months after stopping acupuncture treatment.

Acupuncture treatment consisted of one to three sessions per week that were separated by at least 1 day and lasted for 8 weeks. All of our patients completed a total of 16 treatments. The patients in the acupuncture group underwent the third evaluation 3 months after stopping acupuncture treatment. The flow chart of this study is shown in Figure 1.

### Acupuncture Treatment

The acupuncturists were all well-trained, had been licensed for at least 3 years, and did not participate in any clinical assessments. The needles used for acupuncture were non-recycled and 0.30 × 40 mm in size. In accordance with recommendation from traditional theory, three acupoints were consistently used throughout the complete treatment course, including Bai-Hui (GV20), Shen Guan (77.18), and Yanglingquan (GB34) (20). GV20 is the one of the most important acupoint for treating neuropsychiatric problem in general practitioner (21). Shen Guan is an acupoint of Master Tung. This point is usually for “kidney Qi deficiency.” In the literature review, it was prescribed for sensory complaints such as numbness (22). GB34 has ever been applied acupoint for PD. (23–25).The depth of the acupuncture was approximately 5–10 mm. The needle was inserted until the patient felt soreness, fullness, or another sensation of deqi for 20 s. Needles were then kept at the same position for 30 min before removal.

### MRI Acquisition and Processing

Images were acquired using a 3-Tesla MRI scanner (MAGNETOM Trio A TIM system; Siemens, Erlangen, Germany). The structural images were acquired with a T1-weighted magnetization-prepared rapid acquisition gradient-echo sequence using the following imaging parameters: repetition time/echo time/inversion time = 2,000 ms/2.63 ms/900 ms, 160 axial slices of voxel size = 1 × 1 × 1 mm^3^, and flip angle = 9°. The single-acquisition time was 248 s.

Results for rs-fMRI were acquired with a T2^*^-weighted gradient-echo echo planar imaging sequence using the following parameters: repetition time/echo time = 3,000 ms/45 ms, flip angle = 90°, field of view = 192 × 192 mm, slice thickness = 3 mm, matrix = 64 × 64, and voxel size = 3 × 3 × 3 mm^3^. A total of 160 measurements were acquired. The single-acquisition time was 8 min. Moreover, subjects were instructed to rest with their eyes open and not move.

Image processing was conducted using DPARSF (Data Processing Assistant for Resting-State fMRI) software version 2.2 in MATLAB R2017b (MathWorks, Natick, MA, USA). Individual images were resliced into 2-mm isotropic voxels and spatially smoothed with an 8-mm Gaussian filter; the following recommended procedures were also performed: realignment, coregistration, segmentation, and spatial normalization to Montreal Neurological Institute space (www.mni.mcgill.ca).

After preprocessing, images for each individual were parcellated into 116 regions of interest (ROIs) on the basis of the automated anatomical labeling template. The functional connectivity between ROIs was calculated as the signal similarities in time series by using Pearson's correlation coefficients, which resulted in a functional connectivity matrix with 6,670 connections ([116 × 115]/2) in each subject. Fisher's z-transformation was performed to normalize the correlation coefficients. Visualization of the ROIs with significant changes in connectivity was performed with the BrainNet Viewer (26).

### Statistical Analyses

Analysis of the patient demographic data and Spearman's correlation was performed using IBM SPSS Statistics version 24 software (2016 release; IBM Corp., Armonk, NY, USA). Statistical analysis of the functional connectivity (Wilcoxon signed-rank test and feature selection method of least absolute shrinkage and selection operator) was performed using MATLAB software.

The clinical score changes between the baseline evaluation and after treatment were revealed by performing the Wilcoxon signed-rank test between acupuncture and control groups (Table 2).

**Table 2:** Changes of clinical features between baseline and post-treatment.

	**Acupuncture**	**Control**	**Significance**
UPDRS Total	−7.7 ± 4.5	0.7 ± 4.9	*P* = 0.005
UPDRS I	−0.1 ± 1.3	0.3 ± 2.3	NS
UPDRS II	−2.6 ± 2.0	−0.7 ± 1.0	NS
UPDRS III	−4.1 ± 4.0	0.0 ± 3.8	NS
UPDRS IV	−0.9 ± 2.0	1.1 ± 2.0	NS
BDI-II	1.1 ± 8.8	0.7 ± 8.0	NS
PDSS-2	−1.1 ± 6.8	1.9 ± 5.2	NS
PDQ-39	−5.9 ± 13.4	4.1 ± 10.0	NS
MMSE	−0.1 ± 1.5	0.4 ± 1.3	NS
VAS	−0.7 ± 2.3	−0.1 ± 3.6	NS
KPPS	−10.1 ± 8.0	1.6 ± 9.6	*P* = 0.023

*LEDD, Levodopa Equivalent Dose; UPDRS, Unified Parkinson's Disease Rating Scale; BDI-II, Beck Depression Inventory II; PDSS, Parkinson's Disease Sleep Scale; PDQ, Parkinson's Disease Questionnaire; MMSE, Mini-Mental State Examination; VAS, visual analog scale; KPPS, King's Parkinson's Disease Pain Scale; NS, No significance. The data were expressed as (Mean ± SD)*.

Different contrasts were analyzed by Wilcoxon signed-rank test, including (a) acupuncture group between two-time points (conA), (b) control group between two-time points (conB), (c) the acupuncture vs. the control group at baseline (conC), (d) the acupuncture vs. the control group after the acupuncture treatment (conD).

The correlation between the changes of functional connectivity and changes in neuropsychological scores was also investigated using Spearman's rank correlation. Prior to the correlation test, the least absolute shrinkage and selection operator was used to select significantly relevant ROI links among all functional connections. The statistical significance threshold was set at *P* < 0.05.

## Results

### Demographics and Clinical Results

Sixteen enrolled subjects were divided into two groups. Nine patients completed acupuncture treatment; seven patients who received only the analgesic agent completed two fMRI courses.

At baseline, no significant difference was found between patients with and without acupuncture treatment in terms of pain severity (KPPS, *P* = 0.114; VAS, *P* = 1.000), disease duration (*P* = 0.918), motor severity (UPDRS-III, *P* = 0.351), or levodopa equivalent daily dose (*P* = 0.918), nor was a significant difference observed in psychological aspects (BDI-II, *P* = 0.351; PDSS-2, *P* = 0.091; PDQ-39, *P* = 0.918) or cognitive aspects (MMSE, *P* = 0.091).

After treatment, decreases in KPPS and UPDRS total scores were observed (−46.2%, *P* = 0.023 and −21.6%, *P* = 0.005, respectively), but no significant differences were observed in BDI-II, PDSS-2, PDQ-39, and MMSE scores. The changes of clinical performance are summarized in Table 2.

Six of nine patients in the acupuncture group completed the third clinical evaluation for investigation of the prolonged analgesic effect. Two patients were withdrawn because they insisted on continuing acupuncture therapy. One patient was excluded because of a side effect of antiparkinsonian medications.

### Washout Result

The washout effect was assessed 3 months later in six patients who completed treatment. The KPPS in the washout phase was average 11.8, which showed no significant change with after-treatment KPPS (*P* = 0.625).

### Changes in Functional Connectivity After Acupuncture Therapy

No connection showed significant difference in the conA and conB. Two significant connections were found in the acupuncture vs. control group at baseline (conC). The involved connections were (1) between left middle temporal gyrus and left rectus; and (2) between left rolandic operculum and right biventral lobule of cerebellum. Four significant connections were found in the acupuncture vs. control group after completed the acupuncture treatment (conD). The involved connections were (1) between right flocculus of cerebellum and left superior temporal gyrus; and (2) between right flocculus of cerebellum and left postcentral gyrus; (3) between right flocculus of cerebellum and right postcentral gyrus; (4) between left lobule centralis of cerebellum and left opercular part of inferior frontal gyrus.

The contrast between the acupuncture and the control group in the baseline (conC) was used as a control condition in the analysis of therapeutic effect of acupuncture.

**Figure 2:**
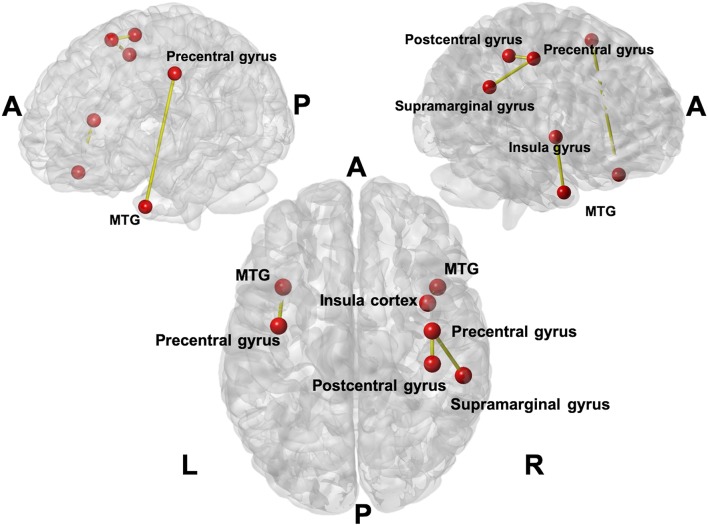
The links with the significant difference in changes between acupuncture and control groups in terms of the changes in functional connectivity between two visits. Four connections (seven anatomical regions were affected) were found with increased connectivity in the acupuncture group. The involved connections included the connection between the Middle Temporal Gyrus (MTG) and precentral gyrus on the left; postcentral gyrus and precentral gyrus on the right; supramarginal gyrus and precentral gyrus on the right, and MTG and insula cortex on the right.

Figure 2 showed the links with significant difference in changes between acupuncture and control groups in terms of the changes of functional connectivity between two visits. Meaningful links were found with increased connectivity in four connections, which were in the left hemisphere between the middle temporal gyrus (MTG) and precentral gyrus and in the right hemisphere between the postcentral gyrus (primary somatosensory cortex; S1) and precentral gyrus, supramarginal gyrus and precentral gyrus, and MTG and insular cortex.

### Correlation With KPPS

**Figure 3:**
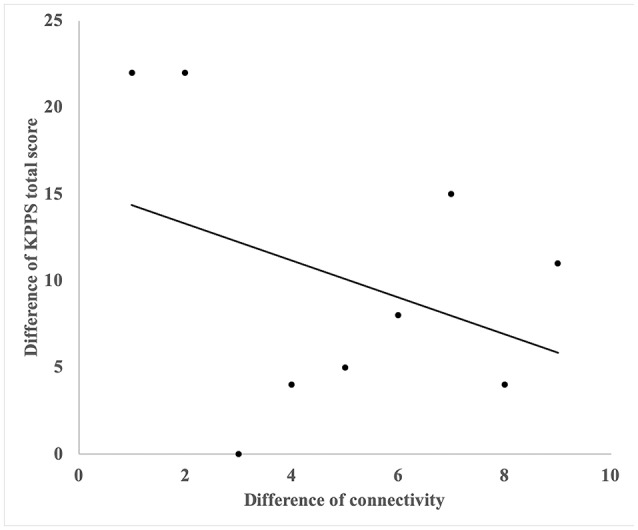
The correlation with reduction of KPPS and changes of functional connectivity of right precentral gyrus and left middle frontal gyrus. A significant correlation was noted between the changes in functional connectivity and changes in total KPPS score in the acupuncture group. The involved connection was between the left middle temporal gyrus and the right precentral gyrus (*R* = −0.698, *P* = 0.037).

A significant correlation was noted between the changes in functional connectivity and changes in total KPPS score in the acupuncture group. The involved connection was between the left MTG and right precentral gyrus (*R* = −0.698, *P* = 0.037) (Figure 3).

In three subdomains of KPPS, significant correlations were found between the reduction in score and changes in functional connectivity. The involved connections in the subdomain of musculoskeletal pain were located between the right posterior cingulate gyrus and right cerebellar lobule IX, between the right caudate nucleus and right transverse temporal gyrus, and between the left cerebellar crus II and right cerebellar lobule IX. In the nocturnal pain subdomain, the involved connection was between the precentral gyrus and the medial orbitofrontal cortex (OFC) in the right hemisphere. In the radicular pain subdomain, the connections were between the right parahippocampal gyrus and left cerebellar lobule VI. Details of significant correlations are listed in Table 3.

**Table 3:** Significant correlations between changes in King's Parkinson's Disease Pain Scale scores (overall and for subdomains) and functional connectivity.

	***P*-value**	**R**
**Total score**		
Right precentral gyrus, left middle frontal gyrus	0.037	−0.698
**Subdomain 1 (musculoskeletal pain)**		
Right posterior cingulate gyrus, right lobule IX of cerebellar hemisphere	0.000	0.932
Right caudate nucleus, right transverse temporal gyrus	0.000	0.932
Left crus II of cerebellar hemisphere, right lobule IX of cerebellar hemisphere	0.000	0.932
**Subdomain 4 (nocturnal pain)**		
Right precentral gyrus, right medial orbitofrontal cortex	0.000	−0.953
**Subdomain 7 (radicular pain)**		
Right parahippocampal gyrus, left lobule VI of cerebellar hemisphere	0.015	−0.772

## Discussion

### Main Finding and Clinical Impact

In the current study, we hypothesized that the mechanism of pain relief through acupuncture in patients with PD, could be provided by altering pain matrix in the brain. In the acupuncture group, no significant difference between two time points might be attributed to the small sample size in our study. However, in the baseline, the difference of functional connectivity between inferior-middle temporal gyrus and rectus could be related to anxiety status in patients with Parkinson's disease (27). Therefore, this contrast in the baseline was used as a control condition. This observation might suggest that the effect of acupuncture is not limited to pain. Our study might provide a new insight to include a comprehensive neuropsychological assessment such as anxiety in patients with Parkinson Disease.

We also observed a modulation of brain connectivity within a pain-related neural network, which might be related to pain relief in the S1, MTG, insular gyrus, and medial orbitofrontal cortex (OFC). We further demonstrated that pain relief by acupuncture is effective and might improve cognitive function. Pain relief could be observed from reduction of KPPS score in the acupuncture group (−46.2%), compared with the control group, although both groups received a conventional analgesic agent (NSAID) during the whole study period.

In addition, a significantly decreased total UPDRS score was noted, which might suggest that the clinical improvement achieved by acupuncture might not be limited to pain relief alone. The prolonged effect of pain relief was observed for at least 3 months after 16 acupuncture courses. In this regard, acupuncture could provide neurologists and patients with an alternative analgesic approach.

### Modulated Connectivity in the Pain Matrix

Patients with PD who received acupuncture treatment showed improvement in KPPS and total UPDRS scores but not in neuropsychological measures (such as MMSE, BDI-II, PDSS-2, and PDQ-39), which might suggest that the fixed acupoint treatment was effective for pain relief mainly in the sensory-discriminative aspect. The pain source can be categorized into affective-motivational and discriminatory aspects that individually form medial and lateral pathways. The medial pathway projects from the medial thalamus to the anterior cingulate cortex and insular cortex and is related to affective-motivational aspects. The lateral pathway projects from the lateral thalamus to the primary and secondary somatosensory cortices and the insular cortex and is related to the sensory-discriminative aspect (12). The sensory-discriminative aspect of pain could be actual pain from the environment, whereas the affective-motivational aspect may potentially be altered by emotions and self-experience (28). The improved UPDRS and KPPS scores but not BDI-II might indicate that the treatment course relieved pain by inhabiting pain itself rather than addressing the emotional reactions of patients with PD.

On the other hand, changes of brain connectivity indicated that the pain relief of acupuncture may be attributable to the increase in connectivity of regions, including S1, MTG, supramarginal gyrus, and insular cortex, which are functionally associated with modulating the general pain pathway and may change the perception of pain from nociceptive receptors to the pain matrix (11).

The S1 is considered to be a major node in the localization and discrimination of pain (29). Chronic pain may be related to the reorganization of S1 (29), which suggests that the S1 cortex might play an important role within the brain networks that mediate chronic pain (29). By contrast, MTG has a connection to the general pain pathway, such as the thalamus and the anterior cingulate and prefrontal cortices (30). The supramarginal gyrus is a somatosensory-associated cortex that may interpret sensory input and is involved in the perception of space and limb location. The insular cortex was also reported to be part of a pain-associated network and reflects the responses of emotions and feelings (31). Considering all of these findings together, the present study might support the hypothesis that the effect of acupuncture may be through a lateral pain pathway (S1, insula) with nociception and then may modulate pain perception by activating other brain regions, such as the MTG.

On the basis of the correlation analysis between the changes of functional connectivity and KPPS scores, we found that the increased connectivity between the left middle frontal gyrus and right precentral gyrus was negatively correlated with total KPPS score. This finding might suggest that stronger connectivity between these regions could be linked to more effective pain relief. The precentral gyrus was reported to be responsible for providing relief from chronic pain (32). Moreover, the middle frontal gyrus is located in the prefrontal cortex, which is part of the pain matrix (33). That is, acupuncture may reduce pain by increasing the connectivity between the precentral gyrus and middle frontal gyrus, which are related to pain relief.

Moreover, we discerned other correlations between changes in functional connectivity and individual domains of the KPPS. In domain 4 of the KPPS, which measures nocturnal pain, we found that the increased functional connectivity of the OFC and precentral gyrus is associated with the reduction of nocturnal pain. Specifically, the medial OFC is connected to the striatum and dopaminergic nuclei (34). Nocturnal pain is mainly related to restless leg syndrome (13), the first-line treatment for which is dopaminergic drugs (35). This may suggest that acupuncture can relieve nocturnal pain in patients with PD through stimulation of the OFC followed by modulation of the dopaminergic pathway.

We also found a persistent pain relief effect of acupuncture in our study. That is, the effect of acupuncture persisted even after stopping acupuncture for 3 months. The persistent effect might further attract new interest in the investigation of acupuncture for the modulation of brain plasticity in the pain matrix (36).

In conclusion, acupuncture could relieve such specific pain in patients with PD by modulating several regions of the brain related to both sensory-discriminative and emotional aspects, especially those correlated with the S1, MTG, insular cortex, prefrontal cortex, and middle frontal gyrus. Moreover, the OFC is a specific region involved in nocturnal pain. The use of rs-fMRI in our study might provide imaging-based evidence for clinical improvement of acupuncture treatment strategy. The current study might increase the confidence of users that acupuncture might be an effective and safe analgesic tool for pain relief in patients with PD.

### Limitations

The present study is limited by a relatively small sample size, but the analgesic effect was prominent in our patients receiving acupuncture treatment. We did not perform sham acupuncture in another group of control subjects, however, acupuncture was shown to be more effective than sham treatment in a meta-analysis of chronic pain (20). This is consistent with both the changes in the clinical evaluation and brain connectivity in our study, which reaffirm the effectiveness of pain relief by acupuncture.

## Data Availability Statement

All datasets generated for this study are included in the manuscript/supplementary files.

## Ethics Statement

The studies involving human participants were reviewed and approved by The ethics committee of the Chang Gung Medical Foundation Institutional Review Board (approval number: 201600710A3C101). The patients/participants provided their written informed consent to participate in this study.

## Author Contributions

S-WY, Y-RW, and J-JW contributed the conception and design of the study. S-WY, S-HL, C-CT, Y-CH, Y-SC, and B-YY organized the database. S-WY and S-HL performed the statistical analysis. S-WY wrote the first draft of the manuscript. S-HL wrote sections of the manuscript. KC provided the appropriate pain evaluation in PD patients. All authors contributed to manuscript revision and read and approved the submitted version.

### Conflict of Interest

The authors declare that the research was conducted in the absence of any commercial or financial relationships that could be construed as a potential conflict of interest.
